# scDecouple: decoupling cellular response from infected proportion bias in scCRISPR-seq

**DOI:** 10.1093/bib/bbae011

**Published:** 2024-02-06

**Authors:** Qiuchen Meng, Lei Wei, Kun Ma, Ming Shi, Xinyi Lin, Joshua W K Ho, Yinqing Li, Xuegong Zhang

**Affiliations:** MOE Key Lab of Bioinformatics & Bioinformatics Division BRNIST, Department of Automation, Tsinghua University, Beijing 100084, China; MOE Key Lab of Bioinformatics & Bioinformatics Division BRNIST, Department of Automation, Tsinghua University, Beijing 100084, China; School of Biomedical Sciences, Li Ka Shing Faculty of Medicine, The University of Hong Kong, Pokfulam, Hong Kong SAR, China; Laboratory of Data Discovery for Health Limited (D24H), Hong Kong Science Park, Hong Kong SAR, China; MOE Key Lab of Bioinformatics & Bioinformatics Division BRNIST, Department of Automation, Tsinghua University, Beijing 100084, China; School of Biomedical Sciences, Li Ka Shing Faculty of Medicine, The University of Hong Kong, Pokfulam, Hong Kong SAR, China; Laboratory of Data Discovery for Health Limited (D24H), Hong Kong Science Park, Hong Kong SAR, China; School of Biomedical Sciences, Li Ka Shing Faculty of Medicine, The University of Hong Kong, Pokfulam, Hong Kong SAR, China; Laboratory of Data Discovery for Health Limited (D24H), Hong Kong Science Park, Hong Kong SAR, China; MOE Key Laboratory of Bioinformatics, Tsinghua University, Beijing 100084, China; IDG-McGovern Institute for Brain Research, Center for Synthetic and Systems Biology, School of Pharmaceutical Sciences, Tsinghua University, Beijing 100084, China; MOE Key Lab of Bioinformatics & Bioinformatics Division BRNIST, Department of Automation, Tsinghua University, Beijing 100084, China; Center for Synthetic and Systems Biology, School of Life Sciences and School of Medicine, Tsinghua University, Beijing 100084, China

**Keywords:** single cell, pooled CRISPR-screening, perturbation effects, statistical model

## Abstract

Single-cell clustered regularly interspaced short palindromic repeats-sequencing (scCRISPR-seq) is an emerging high-throughput CRISPR screening technology where the true cellular response to perturbation is coupled with infected proportion bias of guide RNAs (gRNAs) across different cell clusters. The mixing of these effects introduces noise into scCRISPR-seq data analysis and thus obstacles to relevant studies. We developed scDecouple to decouple true cellular response of perturbation from the influence of infected proportion bias. scDecouple first models the distribution of gene expression profiles in perturbed cells and then iteratively finds the maximum likelihood of cell cluster proportions as well as the cellular response for each gRNA. We demonstrated its performance in a series of simulation experiments. By applying scDecouple to real scCRISPR-seq data, we found that scDecouple enhances the identification of biologically perturbation-related genes. scDecouple can benefit scCRISPR-seq data analysis, especially in the case of heterogeneous samples or complex gRNA libraries.

## INTRODUCTION

With the advancement in single-cell sequencing and CRISPR (clustered regularly interspaced short palindromic repeats) technologies, scCRISPR-seq [1] (single-cell CRISPR sequencing) has emerged as a novel high-throughput gene function profiling method. scCRISPR-seq first leverages CRISPR to perturb a set of genes and then assesses the resulting profiles of each perturbation by single-cell sequencing. There exist multiple scCRISPR-seq protocols, including Perturb-seq [2–4], CROP-seq [5], CRISP-seq [6], Mosaic-seq [7], Spear-ATAC [8] and CRISPR-sciATAC [9]. In a typical scCRISPR-seq protocol, a pool of guide RNAs (gRNAs) targeting different genes are usually packed into lentivirus and then introduced into cells. These gRNAs each introduce perturbation to a subgroup of cells. Then, single-cell sequencing [10–14] is used to capture one or more types of profiles for each cell, such as single-cell RNA sequencing (scRNA-seq) [10, 11], single-cell ATAC sequencing (scATAC-seq) [12, 13] and Cellular Indexing of Transcriptomes and Epitopes by Sequencing (CITE-seq) [14]. These scCRISPR-seq methods enable high-throughput perturbations as well as data-rich read-outs for each perturbation, providing informative data for gene regulation study [2, 15–17], disease target identification [4] and drug development [18].

Obtaining the true effect of each perturbation is a prerequisite for scCRISPR-seq analysis. A primary challenge lies in the inability to directly measure the original expression profile of perturbed cells. In typical experimental settings, a control group, comprising either unperturbed cells or those infected by non-targeting (NT) gRNAs, is introduced to approximate the profiles of cells before perturbations [2, 15, 19]. When regarding the control group as the original state of cells subjected to the perturbation (termed as the perturbation group), the data analysis may suffer from gRNA ‘infected proportion bias’. The infection proportion bias refers to the significant differences in the proportions of cell clusters between the perturbation group and the control group, which may be caused by the inconsistent infection efficiencies of different gRNAs, inherent differences in growth rates among different cell clusters or the sampling bias in sequencing. The infection proportion bias causes the average gene expression level of the control group different from the true original expression profile of the perturbation group. It then causes the traditional perturbation effect estimation by computing the fold change (FC) in the average expression level between the perturbation group and the control group cannot reflect the true cellular response. With the development of scCRISPR-seq technologies, the increasing heterogeneity of samples [4] and the expanding complexity of gRNA libraries [15] intensify the importance of decoupling the cellular response from infected proportion bias.

Here, we developed scDecouple as a solution to solve this problem by employing maximum likelihood estimation. scDecouple leverages a Gaussian mixture model to depict the distribution of gene expression profiles in the principal component (PC) space. Through the expectation–maximization (EM) algorithm, we iteratively approximate the genuine cluster proportion of infected cells along with their cellular responses. We evaluated the performance of scDecouple on both synthetic and real-world datasets. The generation of synthetic data considered various parameter settings, including the distance between clusters and the infection ratio. scDecouple consistently exhibited good performance across different parameter settings. Application to real-world scCRISPR-seq datasets revealed that scDecouple not only provided more precise cellular responses but also enhanced biologically relevant pathway identification and gene ranking. scDecouple facilitates a deeper comprehension of perturbation consequences and paves the way for advancing intricate scCRISPR-seq protocols.

## METHODS

### Mathematical description of infected proportion bias

**Figure 1:**
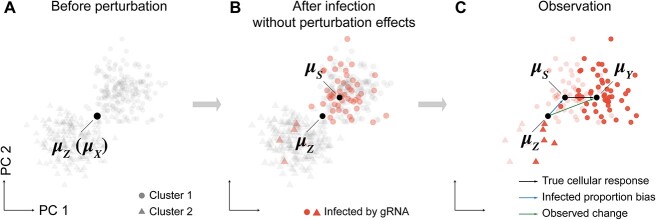
Mathematical description of average expression alteration of the perturbation group. Distributions and average expressions of cells are shown in the feature space. (**A**) Cells before perturbation. (**B**) Cells after infection without the consideration of perturbation effects. (**C**) Cells observed by high-throughput sequencing. Cells in different clusters are represented by different shapes.

We mathematically described the entire experimental process of scCRISPR-seq. Without loss of generality, we assumed that there are two clusters in the experimental cells before perturbation (Figure 1A). Their average expression (${\mu}_X$) is approximately equal to the average expression of cells in the control group (${\mu}_Z$). Due to the infected proportion bias, the average expression of cells may differ from ${\mu}_Z$ even taking no consideration about the expression perturbation caused by gRNAs. We named this average expression as ${\mu}_S$ (Figure 1B). Besides, we assumed that the perturbation brought by a gRNA introduces the same alteration to expression profiles of all cells in the perturbation group in the feature space, leading the average expression of the perturbation group alters from ${\mu}_S$ to the observed one ${\mu}_Y$ (Figure 1C). The observed change between the control and perturbation group is ${\mu}_Y-{\mu}_Z$, which contains the bias caused by infected proportion bias (${\mu}_S-{\mu}_Z$) and the true cellular response to perturbation (${\mu}_Y-{\mu}_S$).

### General workflow of scDecouple

**Figure 2:**
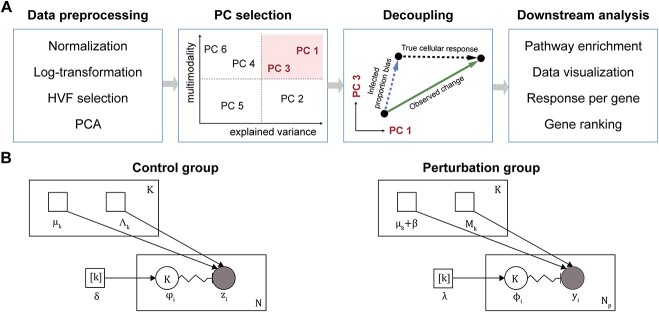
General workflow and probabilistic model of scDecouple. (**A**) Four main steps in scDecouple. (**B**) Plate notes of the GMM probabilistic model of the control and perturbation group. Smaller squares represent fixed parameters. $\left[\mathrm{k}\right]$ means a vector of size K. The circles represent random variables, where filled-in means known values. The directed edges between variables indicate dependencies between the variables. The squiggly line with a crossbar indicates the value selected from upstream variables. The N or K in the corner of the plate indicates that the variables inside are repeated N or K times. Here, ${\varphi}_{i=1\dots N}\sim \mathrm{Categorical}\left(\delta \right)$, ${\phi}_{i=1\dots N}\sim \mathrm{Categorical}\left(\lambda \right)$.

scDecouple comprises four distinct steps: data preprocessing, PC selection, decoupling and downstream analysis (Figure 2A).

During the data preprocessing phase, the gene expression of each cell is first normalized based on its total expression to reduce the technical variance caused by sequencing depths. The expression then undergoes a log transformation to reduce the effects of extremely high-expressed outliers. After, highly variable features (HVFs) of cells in the control group are selected, and the cell-HVF matrix is transformed into the PC space.

The second step is PC selection. The number of cells effectively impacted by a gRNA is limited in scCRISPR-seq, making it hard to estimate a mass of parameters. Thus, we designed a PC selection approach to reduce the number of parameters to be estimated. Our analytical derivations revealed that influence of infected proportion bias is most pronounced in PCs with both high variance and multimodality. scDecouple employs this finding as the selection criteria to select PCs bearing infected proportion bias for further analysis. Details of the step are shown in section PC selection.

After, the decoupling procedure is executed on the selected PCs to disentangle cellular response from infected proportion bias. We projected cells in the perturbation group into the PC space defined by HVFs in the control group. We then constructed two Gaussian mixture models (GMMs) for the control and perturbation groups, respectively. Both the models share the same number of cell clusters, but the parameters within these clusters are different. The EM algorithm is used to iteratively estimate the unknown parameters. With this approach, the genuine cluster proportion of infected cells along with their cellular responses are approximated. Details of the step are shown in sections Decoupling with GMMs and Inferring cellular response with the EM algorithm.

Finally, we integrated several common downstream analyses based on the decoupled results, including pathway enrichment and data visualization. Besides, the gene expression profile can be regenerated from the PC space, and a ranked list of perturbation-related genes is provided to help explore the cellular response of any gene to a perturbation.

#### PC selection

Despite the large number of sequenced cells, the number of cells effectively impacted by a gRNA is usually small in scCRISPR-seq due to high complexity of gRNA pools and limited gRNA efficiency. For example, the median of cells captured by a single gRNA is 121 in a K562 scCRISPR-seq experiment [15] and 83 in a Jurkat cell experiment [5]. Reducing the number of parameters to be estimated is crucial to amplify estimation accuracy. To address this issue, we classified PCs based on their distribution of data. We divided PCs into two types. The first type of PCs contains high-variance data and multimodal data distribution. Other PCs are classified as the second type, which contain low-variance data or unimodal data distribution. We proved in the following context that only the first category of PCs is susceptible to the bias of infected proportion when calculating perturbation effects. Therefore, the decoupling step can be performed only on the first category of PCs to improve the power of fitting. Below is the derivation of the formulas and the criteria for classifying the two types of PCs.

We first derived the relationship between the observed change, cellular response and infected proportion bias on each PC. We denoted the expression of cell $i$ in the control group on this PC as ${z}_i$ and the expression of cell $j$ in the perturbation group on this PC as ${y}_j$. The observed change (OC) is the difference between two groups:


(1) \begin{equation*} \mathrm{OC}=\frac{1}{N_P}{\sum}_{j=1}^{N_P}{y}_j-\frac{1}{N_c}{\sum}_{i=1}^{N_c}{z}_i \end{equation*}

where ${N}_P$ and ${N}_C$ represent the number of cells in the perturbation and control group, respectively. We assumed that cells in the control group have $K$ clusters, and the average expression profile on this PC and proportion of cluster $k$ is ${\mu}_k$ and ${\delta}_k$, respectively. After perturbation, we assumed that the cluster number maintains as $K$ but the proportion of cluster $k$ in the perturbation group changes to ${\lambda}_k$. There are


(2) \begin{equation*} {\sum}_{k=1}^K{\delta}_k={\sum}_{k=1}^K{\lambda}_k=1 \end{equation*}

Besides, we assumed that all perturbed cells share a similar response $ \beta $. Thus, 


(3) \begin{equation*} \mathrm{OC}={\sum}_{k=1}^K{\lambda}_k\left({\mu}_k+\beta \right)-{\sum}_{k=1}^K{\delta}_k{\mu}_k=\beta +{\sum}_{k=1}^K\left({\lambda}_k-{\delta}_k\right){\mu}_k \end{equation*}

In this equation, the first term represents the cellular response, and second term represents the infected proportion bias.

The infected proportion bias approaches zero under two specific conditions. One condition is that the data on this PC follow a unimodal distribution ($K=1$, and thus ${\lambda}_1={\delta}_1=1$)$.$ Another condition is that the data variance explained by this PC is low, and thus


(4) \begin{equation*} {\mu}_k\approx \overline{\mu}=\frac{1}{N_c}{\sum}_{i=1}^{N_c}{z}_i \end{equation*}

(5) \begin{equation*} \mathrm{OC}\approx \beta +\overline{\mu}{\sum}_{k=1}^K\left({\lambda}_k-{\delta}_k\right)=\beta \end{equation*}

Therefore, in cases where PCs exhibit a unimodal distribution or low explained variance, the observed change is equal or approximately equal to real cellular response. In such scenarios, there is little space for improvement through decoupling. Decoupling is primarily required for PCs that exhibit both multimodality and high explained variance.

The explained variance of a PC is calculated during PC analysis (PCA). The multimodality score of a PC is measured by Hartigan’s dip test [20], which quantifies the maximum difference between the empirical distribution and the best-fitting unimodal distribution. PCs that are both multimodal and with high explained variance are selected for decoupling. For any remaining PC, OC calculated by Equation (1) is directly regarded as the cellular response.

#### Decoupling with GMMs

We used GMMs to describe the gene expression of cells in the control or perturbation group on all selected PCs. The density function of the control group is


(6) \begin{align*} p(z)&={\sum}_{k=1}^K{\delta}_k\mathcal{N}\left(z|{\mu}_k,{\varLambda}_k^{-1}\right)\nonumber \\ &={\sum}_{k=1}^{\mathrm{K}}{\delta}_k{\left|\frac{\varLambda_k}{2\pi}\right|}^{\frac{1}{2}}\exp \left[-\frac{1}{2}{\left(z-{\mu}_k\right)}^T{\varLambda}_k\left(z-{\mu}_k\right)\right] \end{align*}

Here, ${\mu}_k$ and ${\varLambda}_k$represent the expectation (mean) and precision (inverse of variance) of cell cluster $k$ in the control group on all selected PCs, respectively.

We applied the Bayesian information criterion (BIC) to determine the number of clusters $K$. The BIC is calculated using the following formula:


(7) \begin{equation*} BIC(K)=-2\max \log \left({L}_K\right)+\left(\log n\right)d(K) \end{equation*}

where ${L}_K$ is the likelihood of the model with $K$ clusters, $d(K)$ is the number of parameters for the model with $K$ clusters and $n$ is the sample size.

The density function of the perturbation group is


(8) \begin{align*} p(y)&={\sum}_{k=1}^K{\lambda}_k\mathcal{N}\left(y|{\mu}_k+\beta, {M}_k^{-1}\right) \nonumber \\ & ={\sum}_{k=1}^K{\lambda}_k{\left|\frac{M_k}{2\pi}\right|}^{\frac{1}{2}}\exp \left[-\frac{1}{2}{\left(y-\left({\mu}_k+\beta \right)\right)}^T{M}_k\left(y-\left({\mu}_k+\beta \right)\right)\right] \end{align*}

Here, ${M}_k$represents the and precision of cell cluster $k$ in the perturbation group, and $\beta$ represents the cellular response on all selected PCs. Figure 2B shows the plate notes of the two GMMs for the control and perturbation group, separately.

#### Inferring cellular response with the EM algorithm

The likelihood function for all observed data is


(9) \begin{align*} L\left(\theta |z,y\right)&=L\left(\delta, \mu, \varLambda, \lambda, \beta, M|z,y\right) \nonumber \\ &={\prod}_{j=1}^{N_C}p\left({z}_j|\delta, \mu, \varLambda \right){\prod}_{i=1}^{N_P}p\left({y}_i|\lambda, \beta, M\right)={L}_c{L}_P \end{align*}

The first component represents the likelihood of the control group (${L}_c$), and the second represents the likelihood of the perturbation group (${L}_P$).

The EM algorithm is first employed to maximize ${L}_c$ and estimate parameters $\delta, \mu, \varLambda$:


(10) \begin{align*} \max{L}_c & =\max{\prod}_{i=1}^{N_c}p\left({z}_i|\delta, \mu, \varLambda \right) \nonumber \\ &=\max{\prod}_{i=1}^{N_c}{\prod}_{k=1}^K{\left[p\left({u}_i=k|\delta \right)p\left({z}_i|{u}_i=k,{\mu}_k,{\varLambda}_k^{-1}\right)\right]}^{I\left({u}_i=k\right)} \end{align*}

where ${U}_i$ indicates the component of ${Z}_i$ and each ${U}_i$ has $K$ possible outcomes. In other words,


$$ U\sim Multinomial\ train\left(K,\delta \right) $$

The EM algorithm is then used to maximize ${L}_P$:


(11) \begin{equation*} \max{L}_P=\max{\prod}_{j=1}^{N_P}p\left({y}_j|\lambda, \mu, \beta, M\right) \end{equation*}

As $\mu$ has already been estimated in the previous step, the iterative EM algorithm is used to estimate$\lambda, \beta, M$. We use ${V}_i$ to indicate the component of ${Y}_i$.


$$V\sim Multinomial\ trail\left(K,\lambda \right)$$

For the expectation step (E-step):


(12) \begin{align*} &\mathrm{E}\left[{V}_j=k|\hat{\lambda},\hat{\beta}\right] \nonumber \\&=\frac{\lambda_k{\left|{M}_k\right|}^{\frac{1}{2}}\exp \left\{-\frac{1}{2}{\left({y}_j-\left({\hat{\mu}}_k+{\hat{\beta}}_k\right)\right)}^T{M}_k\left({y}_j-\left({\hat{\mu}}_k+{\hat{\beta}}_k\right)\right)\right\}}{\sum_{l=1}^K{\mathrm{\lambda}}_l{\left|{M}_l\right|}^{\frac{1}{2}}\exp \left\{-\frac{1}{2}{\left({y}_j-\left({\hat{\mu}}_l+{\hat{\beta}}_l\right)\right)}^T{M}_l\left({y}_j-\left({\hat{\mu}}_l+{\hat{\beta}}_l\right)\right)\right\}} \end{align*}

For the maximum step (M-step):


(13) \begin{align*} & {\hat{\mathrm{\lambda}}}_k=\frac{1}{N_P}\sum_{j=1}^{N_P}\mathrm{E}\left[{V}_j=k\right] \nonumber \\ &\hat{\beta}=\frac{1}{K}{\sum}_{k=1}^K\frac{\sum_{j=1}^{N_P}\mathrm{E}\left[{V}_j=k\right]\left({y}_j-{\hat{\mu}}_k\right)}{\sum_{j=1}^{N_P}\mathrm{E}\left[{V}_j=k\right]} \nonumber \\&{\hat{M}}_k=\frac{\sum_{j=1}^{N_P}\mathrm{E}\left[{V}_j=k\right]\left({y}_j-\left({\hat{\mu}}_k+{\hat{\beta}}_k\right)\right){\left({y}_j-\left({\hat{\mu}}_k+{\hat{\beta}}_k\right)\right)}^T\ }{\sum_{j\in{N}_P}\mathrm{E}\left[{V}_j=k\right]} \end{align*}

The E-step and M-step are iteratively applied till convergence. In certain cases, we can choose to fix ${\hat{M}}_k$ as ${\hat{\varLambda}}_k$ to make the shape of cell clusters in the perturbation group similar to those in the control group.

## RESULTS

### Performance of scDecouple on synthetic data

**Figure 3:**
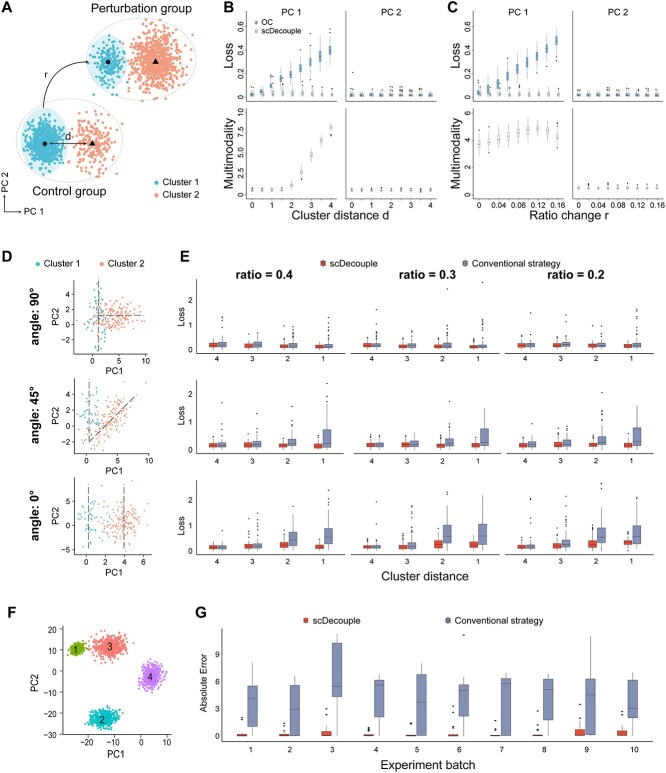
Schematic diagram of synthetic data and performance of different methods on synthetic data. (**A**) The illustration of synthetic data. (**B**) Performance of different methods on synthetic data with different cluster distances $d$. (**C**) Performance of different methods on synthetic data with different ratio changes $r$. (**D**) The distribution of cells in the perturbation group with different covariance matrix angles. The dashed lines represent the direction of maximum variance in two clusters. (**E**) Performance of different methods on synthetic data with different cluster angles, gRNA infection efficiency and cluster distance. Each block represents the gRNA infection efficiency for cluster 1 from 0.4 to 0.1, while the ratio of cluster 2 was fixed at 0.3. The *x*-axis represents the distance between two clusters. Different columns represent different covariance matrix angles. (**F**) The distribution of cells in the perturbation group with multiple clusters. Different colors represent different clusters. (**G**) Performance of different methods on synthetic multi-cluster data with different gRNA infection efficiencies. The *x*-axis represents different experimental batches with varied gRNA efficiencies.

We conducted a series of simulation experiments to demonstrate the performance of scDecouple in decoupling cellular response from infected proportion bias. We first generated a synthetic dataset. We randomly generated 1000 NT cells with two-dimensional PCs as the control group. These cells followed a two-cluster GMM (Figure 3A), whose bimodality concentrates on PC 1. We used the same probability model to randomly generate the initial states of cells in the perturbation group and added perturbation effects on these cells with the assumption that all cells exhibit similar responses to the perturbation in the PC space. According to Equation (3), when the cluster number is 2, ${\mu}_1+{\mu}_2={\delta}_1+{\delta}_2=1$, and thus the infected proportion bias of any PC in the scenario of two clusters is


(14) \begin{align*} {\sum}_{k=1,2}\left({\lambda}_k-{\delta}_k\right){\mu}_k&=\left({\lambda}_1-{\delta}_1\right){\mu}_1+\left({\lambda}_2-{\delta}_2\right){\mu}_2\nonumber \\ &=\left({\lambda}_1-{\delta}_1\right){\mu}_1+\left[\left(1-{\lambda}_1\right)-\left(1-{\delta}_1\right)\right]{\mu}_2 \nonumber \\&=\left({\lambda}_1-{\delta}_1\right)\left({\mu}_1-{\mu}_2\right) \end{align*}

The bias was linearly correlated with two components: the distance $d$ between two clusters and ratio change $r$ of cluster 1 between two groups:


(15) \begin{align*} & d=\mathrm{abs}\left({\mu}_1-{\mu}_2\right) \nonumber \\& r=\mathrm{abs}\left({\lambda}_1-{\delta}_1\right) \end{align*}

By varying the values of $d$ and $r$, we systematically controlled the magnitude of infected proportion bias. For each parameter combination, we calculated OC as the results of naive analysis and applied scDecouple to estimate real cellular response. We compared the results of these methods with ground truth. We also evaluated the effectiveness of Hartigan’s dip test as the metric for assessing the inherent multimodality of the control group.

We first fixed the ratio change $r=0.1$ and varied the cluster distance $d$ (Figure 3B) and then fixed $d=3$ and varied $r$ (Figure 3C). We conducted 100 simulations for each parameter setting. When $d$ or $r$ was small, OC and the scDecouple results both exhibited low losses on PC 1 (Figure 3B and C). However, with the increase of data multimodality or ratio change, estimation by OC showed higher losses, whereas scDecouple continued to perform well. In the PC 2 dimension where the data distribution is unimodal, both methods showed similar results (Figure 3B and C). Besides, our results demonstrated that Hartigan’s dip test in scDecouple exhibited high sensitivity to changes in multimodality (Figure 3B and C).

The results revealed that when facing cellular heterogeneity and uneven gRNA sampling across different cell clusters, OC can hardly be regarded as cellular response as it is affected by infected proportion bias. In contrast, scDecouple decouples these intertwined factors and consistently produces low-error estimation outcomes, irrespective of the degree of infected proportion bias.

We conducted another two simulations to compare scDecouple with a conventional strategy. The procedures of conventional strategy consists of clustering and annotation in control and perturbation groups, respectively, and mapping clusters in two groups to calculate the perturbation effects. Here, we utilized GMM estimation to compute the cluster centers and annotated cell clusters based on their rankings of cluster centers on the first PC. Then, we calculated the average change in cluster centers before and after perturbation as perturbation effects.

The first experiment simulated a control group with two clusters with varied covariance matrix angles, cluster distances and gRNA infection efficiencies. The covariance matrix angle is the angle between the axis with the maximum variance of cluster 1 and cluster 2 (Figure 3D). A low covariance matrix angle will result in an ambiguous separation of two clusters. The perturbation effects were simulated to be identical for both clusters. For each parameter combination, we conducted 100 times of experiments by random sampling. We employed the conventional strategy and scDecouple to infer perturbation effects, respectively. As shown in Figure 3E, we found that scDecouple got lower errors than the conventional strategy. The performance of the conventional strategy is less stable, especially when distance between the two clusters decreases or the number of cells captured by gRNA diminishes. This situation becomes more severe as the cluster angle decreases to 0. In these scenarios, the conventional strategy may gain more errors when clustering the control and perturbation groups separately. On the contrary, scDecouple learns the cluster information from the control group, which can be used to assist the clustering of the perturbation group. This particularly matters for current scCRISPR-seq data, which usually show low gRNA efficiency and few cells in the perturbation group.

The second simulation experiment addressed a multi-cluster scenario. We randomly generated four clusters following GMMs on a two-dimensional PC space as the control group, each with distinct means and covariance matrices, as shown in Figure 3F. We randomly generated 10 gRNAs, each having a unique array of infection efficiency for different clusters. For each parameter, we conducted 50 experiments by randomly generating data. We compared the performance of scDecouple and the conventional strategy on these data (Figure 3G). The results showed that when experimental data involve multiple clusters, the use of a single marker in the conventional strategy cannot consistently capture the correct cluster correspondence between the control and perturbation groups. In contrast, scDecouple considers the relative distances between clusters during parameter estimation and thus ensures the correct correspondence between clusters.

From the simulation experiments, it is evident that scDecouple consistently shows more stable estimation results and lower errors compared with other strategies. Moreover, scDecouple does not rely on the definition of cluster markers or additional steps to solve for the correspondence between clusters before and after perturbation, which simplifies the process of perturbation effect estimation.

### Performance of scDecouple on simulated biologically derived genome-wide data

**Figure 4:**
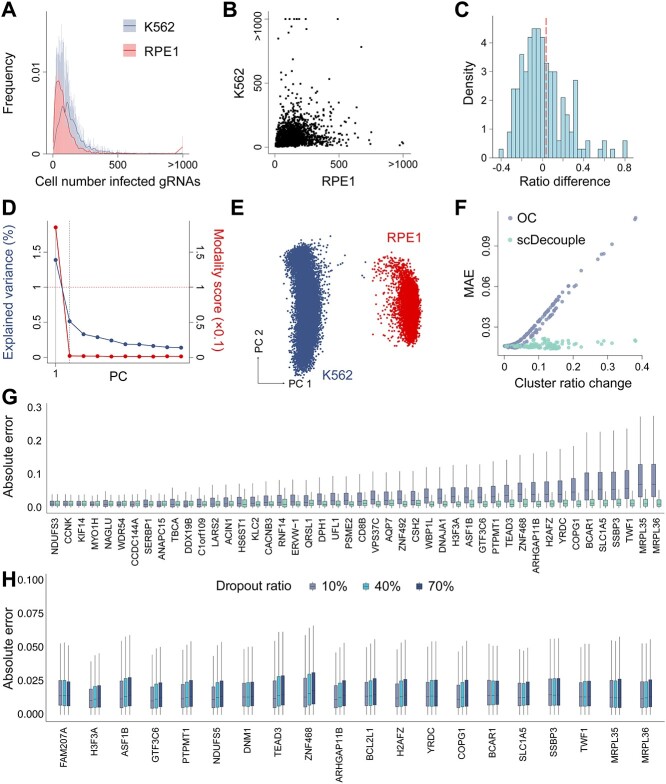
Performance of different methods on simulated genome-scale scCRISPR-seq data. (**A**) The distribution of the cell number infected by different gRNAs in different cell lines. Solid lines show the smoothing spline fitting curve with a 0.5 smoothing parameter. (**B**) The cell number infected by the same gRNA between different cell lines. (**C**) The gRNA infection ratio difference between the RPE1 and K562 cluster in the perturbation group. The dashed line represents the infection ratio difference in the control group. (**D**) The explained variance and multimodality score of each PC. Dashed lines represent thresholds for identifying high variance or multimodality. (**E**) The distribution of cells in PC 1 and PC 2. (**F**) Cluster ratio changes and MAEs of cell response estimations on PC1. Each dot represents a gRNA. (**G**) Absolute error of cell response estimations in gene expression profiles. One sample represents a gene in the gene expression profile. The *x*-axis represents gRNA targets, sorted by cluster ratio changes. (**H**) Absolute errors of cell response estimations in gene expression profiles on the top 20 gRNAs.

We conducted simulations using real scCRISPR-seq data to further evaluate the performance of scDecouple. We collected a scCRISPR-seq dataset from a genome-wide Perturb-seq study [15] and selected the experimental data from two distinct cell lines, K562 and RPE1. We mixed these data to simulate two cell clusters in a single experiment. We found that the cell number infected by different gRNAs varied in both cell lines (Figure 4A). The correlation of the cell number infected by the same gRNA between different cell lines is notably low (Pearson’s correlation coefficient = 0.155, Figure 4B), suggesting significant infected proportion bias between cell clusters. The difference between the infection ratio of the RPE1 cluster and the K562 cluster was varied and differed from the control group (Figure 4C).

We applied scDecouple on the simulated data. We found that PC 1 showed both the highest explained variance and the highest multimodality score among all PCs (Figure 4D and E). Thus, we performed decoupling on PC 1 with the cell cluster number $K$ = 2 and regarded OC as the cellular response for all other PCs. For the perturbation group, we selected 168 editing loci that were designed in both cell line groups and showed similar responses in both cell types (the difference of cellular response on PC 1 less than 5). We took the mean responses (log FC) of the two cell lines on PC 1 as true cellular response and used the mean absolute error (MAE) to assess the accuracy of cell response estimations. We found that scDecouple can better estimate true cellular response than OC, especially when cells infected by a gRNA displayed a substantial cluster ratio change (Figure 4F). We then randomly selected several gRNAs and generated the cellular response of these gRNAs from the PC space to gene expression. We found that responses calculated by scDecouple showed lower MAEs than OC, especially when ratio changes were large (Figure 4G). In general, the results showed that scDecouple revealed more accurate cellular responses and reduced the error introduced by infected proportion bias.

We then explored the impact of batch effects between different samples on scDecouple. We introduced different levels of batch effects by utilizing 10%, 40% and 70% dropout rates in the K562 data. Then, we evaluated the performance of scDecouple by calculating the absolute error of each experiment. We visualized the performance of scDecouple in top 20 gRNAs with the highest OC errors (Figure 4G), and the results demonstrated that scDecouple is not influenced by batch effects and consistently delivers robust estimation results (Figure 4H).

### Applying scDecouple on real scCRISPR-seq data

**Figure 5:**
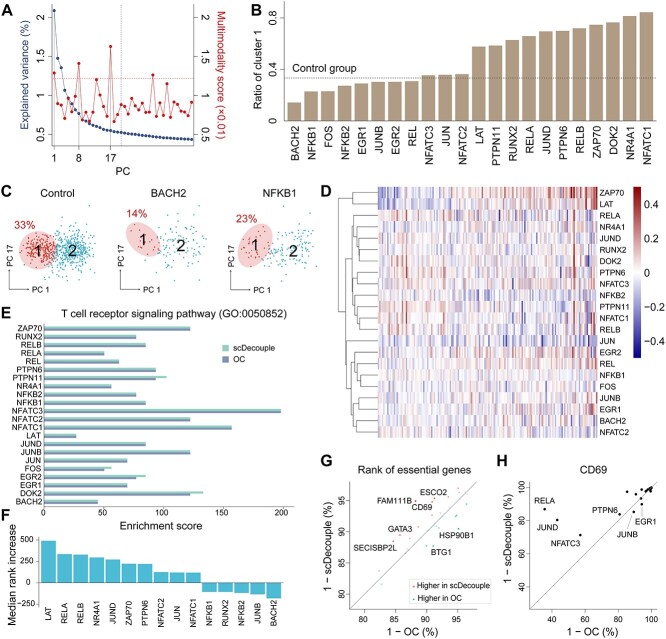
The performance of scDecouple on real scCRISPR-seq data. (**A**) The explained variance and multimodality score of each PC. Dashed lines represent thresholds for identifying high variance or multimodality. (**B**) The ratios of cluster 1 across all targets. (**C**) The distribution of cells in PC 1 and PC 17. The circles highlight the cells in cluster 1. (**D**) Cellular response estimations of all targets in the expression of all TCR pathway–related signature genes. (**E**) The enrichment scores of differential genes in the TCR signaling pathway for each target. (**F**) The median rank increase of gRNAs with similar enrichment scores using scDecouple versus OC. We used Fisher’s exact test to evaluate the overlap between differential genes and TCR pathway–related signature genes. (**G**) Average gene ranking of top 60 genes from the TCR pathway signature genes across all gRNAs. Each dot represents one signature gene. The black line represents $y=x$. The color of each dot represents the change in ranking relative to all gRNAs. The transparency of each dot corresponds to the absolute value of the ranking change, where lower transparency indicates a smaller absolute change. (**H**) Ranking of CD69 in the absolute values of cellular responses calculated by OC or scDecouple. Each dot represents one target. The black line represents $y=x$.

We applied scDecouple to a real scCRISPR-seq dataset targeting T cell receptor (TCR)–related genes in human Jurkat cells [5], which have been reported to have heterogeneity [21, 22]. Each gene was targeted by three gRNAs. We selected 22 target genes each with over 60 infected cells. We considered the cells affected by gRNAs targeting the same gene as one perturbation group. We then got 22 perturbation groups along with NT gRNAs as the control group for downstream analysis. We performed data preprocessing on the data and then transformed the data into the PC space of the control group based on the top 700 highly variable genes. We identified three PCs with both high explained variances and high multimodality scores (Figure 5A). We performed decoupling on these three PCs with the cell cluster number $K$ = 2. We found that the estimated cell cluster proportion for each target varied (Figure 5B), and the inferred clusters showed distinct differences in the PC space (Figure 5C). We generated the cellular response of all targets from the PC space to gene expression obtained the average response of a gene by averaging the response of all gRNAs targeting this gene. We visualized the responses of TCR pathway signature genes defined in the original publication [5] (Figure 5D). The results showed that knockouts of some genes caused pathway activation, while others led to pathway inhibition.

We then compared the cellular response estimated by OC and scDecouple. We identified genes with the top 1500 absolute values of cellular responses as differential genes for the OC and scDecouple results, respectively, and then calculated the enrichment score of the TCR signaling pathway for each target by Fisher’s exact test (Figure 5E). The results showed that scDecouple achieved similar or higher enrichment scores than OC. We quantified the comparison results of pathway enrichment between scDecouple and OC from the perspective of gene rankings. For four gRNAs with improved enrichment score versus OC (PTPN11, FOS, EGR2 and DOK2), we found that scDecouple identified more TCR pathway–related differential genes, leading to an average increase of 280 gene rankings. We also investigated the effect of scDecouple on TCR pathway–related genes for other gRNAs that achieved similar enrichment scores with OC. We compared the differences in cellular response rankings of differential genes between the two methods and focused on TCR-related genes with ranking differences greater than 100. As shown in Figure 5F, for a majority gRNAs, using scDecouple significantly improved the ranking of TCR-related genes, with an average increase of 125 in ranking across all gRNAs.

We further investigated whether scDecouple helps biological discovery. We selected the top 60 genes from the TCR pathway signature genes according to the original study [5] and calculated the average rank of these genes in the absolute values of cellular responses calculated by OC and scDecouple, respectively. We found that these genes showed higher rank in scDecouple results (Figure 5G). We specifically examined CD69, a widely recognized early activation marker of the TCR pathway [23, 24]. The results demonstrated that CD69 rankings were greatly improved for most targets (Figure 5H). The most significant improvement showed in the perturbation group targeting RELA, which was reported to be highly associated with CD69 [25–27]. All the results indicated that scDecouple can benefit the analysis of real scCRISPR-seq data with more precise gene ranking and more accurate gene regulation identification.

## DISCUSSION

We initially formalized the process of cellular response estimation in scCRISPR-seq and derived mathematical equations to deduce estimation bias induced by infected proportion bias. To reduce the influence of infected proportion bias, we introduced scDecouple, a method aimed at unraveling observed changes in scCRISPR-seq data. Our approach focuses on decoupling cellular response from infected proportion bias through maximum likelihood estimation. We validated the efficacy of scDecouple through a series of simulations and its application to a real dataset. scDecouple yielded more enriched pathways and improved the ranking of perturbation-related genes. The good performance of scDecouple is mainly attributed to its estimation of the actual proportions of clusters for infected cells in perturbation groups.

With the development of technology, scCRISPR-seq with more complex gRNA libraries will be developed and applied to populations of more heterogeneous cells. As a result, reducing infected proportion bias will be more important and necessary in the analysis of scCRISPR-seq. Besides, double-strand breaking induced by CRISPR knockout may cause cell state arrest, which will introduce additional bias to cell cluster proportion especially in studies related to cell cycle, senescence or aging. scDecouple can help to discard these impacts and focus on the cellular responses we concern about. Also, unlike the simple approach of conducting separate differential expression analyses for different clusters, the uniqueness of scDecouple lies in its ability to preserve the shape and variance of individual clusters before and after perturbation. This is particularly valuable when dealing with data that exhibit multiple clusters and when there is no clear cluster structure in continuous data, as our method does not rely on a pre-defined set of clusters and analyzes the entire dataset to reach the maximum likelihood.

We have built an R package for scDecouple, offering a comprehensive suite of functionalities for streamlined scCRISPR-seq data analysis. The package comes equipped with an integrated one-click data preprocessing pipeline, infected proportion bias quantification, cellular response estimation and downstream analytical tools. Additionally, the library provides many visualization options, including PC plots, PC variance and multimodality visualizations, cellular response heatmaps and Gene Ontology enrichment plots. Moreover, the library supports both step-by-step execution and parameter customization to meet the diverse needs.

As the first method designed to handle infected proportion bias in scCRISPR-seq, scDecouple can be further optimized. Currently, scDecouple operates under three key assumptions: cells following GMM within the PC space, same cell clusters between NT and perturbation groups and similar cellular responses across cell clusters. The first assumption holds well in certain cell lines and tissues, but it might not remain in intricate systems or disease tissues, such as tumors. We can expand scDecouple by considering alternative data representation methods or statistical models to handle more complex tissues and systems. The second assumption might not hold true in extreme cases, such as when some rare cell types are completely absent from infection. In such a situation, the number of clusters in the control and perturbation group could differ. We plan to incorporate additional information to address this problem, such as including marker genes of cell clusters to link clusters in the perturbation group to those in the control group. The third assumption guarantees that scDecouple is suitable for cells with moderate heterogeneity, which is validated in this study by applying scDecouple on real scCRISPR-seq data. In the future, we intend to integrate more intricate models, such as deep learning, with scDecouple to handle more complex experimental data.

**Key Points**

- We found that infected proportion bias distorts the genuine cellular response to perturbation in scCRISPRseq data.
- We purposed scDecouple to decouple the cellular response from infected proportion bias by utilizing maximum likelihood estimation.
- scDecouple improves estimation of perturbation effects in both simulation experiments and real scCRISPRseq data.
- The scDecouple R package offers decoupling process and streamlines scCRISPR-seq data analysis.

## Data Availability

We only use public datasets in this study. The K562 and RPE1 datasets employed for simulations were obtained through accession code GSE92872. The Jurkat cell dataset was downloaded from the website https://gwps.wi.mit.edu. All codes of this study, including the R package of scDecouple, simulation experiments and real scCRISPR-seq data analysis, are available on GitHub via the following link: https://github.com/MengQiuchen/scDecouple.

## Author Biographies

**Qiuchen Meng** is a PhD candidate at the Department of Automation, Tsinghua University. His research interests include single-cell bioinformatics and machine learning.

**Lei Wei** is a research assistant professor at BNRIST, Tsinghua University. His research interests include single-cell bioinformatics, synthetic biology and machine learning.

**Kun Ma** is an MPhil candidate at the School of Biomedical Sciences, The University of Hong Kong. Her research interests include spatial transcriptomic methods and single-cell transcriptomic analysis.

**Ming Shi** earned his PhD degree at the Department of Automation, Tsinghua University. His research interests include statistical learning and bioinformatics.

**Xinyi Lin** is a PhD student in the School of Biomedical Sciences, The University of Hong Kong. Her research interests include statistical modeling and single-cell multi-omics analysis.

**Joshua Wing Kei Ho** is an associate professor in the School of Biomedical Sciences, The University of Hong Kong. His research interests include single-cell data analysis and microbiome functional systems biology.

**Yinqing Li** is an associate professor at the School of Pharmaceutical Sciences, Tsinghua University. His research interests include machine learning, single-cell multi-omics analysis and cell editing.

**Xuegong Zhang** is the director of the Bioinformatics Division, BNRIST and a professor at the Department of Automation, Tsinghua University. His research interests include pattern recognition, machine learning and bioinformatics.
